# The Effects of an Olive Fruit Polyphenol-Enriched Yogurt on Body Composition, Blood Redox Status, Physiological and Metabolic Parameters and Yogurt Microflora

**DOI:** 10.3390/nu8060344

**Published:** 2016-06-03

**Authors:** Kalliopi Georgakouli, Anastasios Mpesios, Demetrios Kouretas, Konstantinos Petrotos, Chrysanthi Mitsagga, Ioannis Giavasis, Athanasios Z. Jamurtas

**Affiliations:** 1 Department of Physical Education and Sport Science, University of Thessaly, Karies, Trikala 42100, Greece; kgeorgakouli@gmail.com; 2 Department of Nutrition and Dietetics, Technological Educational Institute of Thessaly, Karditsa 43100, Greece; 3 Department of Kinesiology, Institute for Research and Technology Thessaly, Trikala 42100, Greece; 4 Department of Biochemistry and Biotechnology, University of Thessaly, Larissa 41221, Greece; bes_tas@yahoo.gr (A.M.); dkouret@uth.gr (D.K.); 5 Polyhealth S.A., 3rd klm Larisa-Tyrnavos, Larisa 41500, Greece; petrotos@teilar.gr; 6 Department of Food Technology, Technological Institute of Thessaly, Karditsa 43100, Greece; mitsagga.chrisanthi@hotmail.com (C.M.); igiavasis@teilar.gr (I.G.)

**Keywords:** olive polyphenols, yogurt, dairy, health, lipids, lactic acid bacteria, Medoliva©

## Abstract

In the present study we investigated the effects of an olive polyphenol-enriched yogurt on yogurt microflora, as well as hematological, physiological and metabolic parameters, blood redox status and body composition. In a randomized double-blind, crossover design, 16 (6 men, 10 women) nonsmoking volunteers with non-declared pathology consumed either 400 g of olive fruit polyphenol-enriched yogurt with 50 mg of encapsulated olive polyphenols (experimental condition—EC) or 400 g of plain yogurt (control condition—CC) every day for two weeks. Physiological measurements and blood collection were performed before and after two weeks of each condition. The results showed that body weight, body mass index, hip circumference and systolic blood pressure decreased significantly (*p* < 0.05) following the two-week consumption of yogurt regardless of condition. A tendency towards significance for decreased levels of low density lipoprotein (LDL) cholesterol (*p* = 0.06) and thiobarbituric acid reactive substances (*p* < 0.05) following two weeks of polyphenol-enriched yogurt consumption was observed. The population of lactic acid bacteria (LAB) and production of lactate in yogurt were significantly enhanced after addition of olive polyphenols, contrary to the population of yeasts and molds. The results indicate that consumption of the polyphenol-enriched yogurt may help individuals with non-declared pathology reduce body weight, blood pressure, LDL cholesterol levels and lipid peroxidation, and promote growth of beneficial LAB.

## 1. Introduction

Yogurt is a dairy product that has been a part of the human diet for thousands of years. Yogurt is classified as a food of high nutritional value due to its relatively low fat content in combination with its high protein content, as well as vitamins of the B complex, phosphorus, magnesium and potassium. However, nutritional value of different yogurts may significantly vary depending on the type of milk and straining process.

Yogurt (and other dairy product) consumption has been shown to have various beneficial effects on many aspects of human health, including blood pressure (BP) and low density lipoprotein (LDL) cholesterol reduction, muscle building and prevention of various metabolic diseases [1]. Concerning gastrointestinal health, yogurt contains cultures of lactic acid bacteria that improve digestive system function through their positive impact on gut microflora, bowel transit and gastrointestinal immune responses [2]. Finally, there are many reports indicating that yogurt helps with body weight management possibly in part due to satiety and improved gut function caused by probiotic bacteria [3].

Polyphenols are chemical compounds of plant origin that act as antioxidants and are known for their potential beneficial effects on health. Although some bioactive substances have been added to yogurt in order to enhance the health outcomes of conventional yogurt, there is no report in the literature on the possible health effects of a yogurt enriched with polyphenols. Recent studies showed that olive polyphenols may enhance the growth of several lactic acid bacteria *in vitro* [4] and also accelerate the drop of pH during yogurt fermentation [5]; however, it is not clear whether olive polyphenols may affect human metabolism by inducing the growth of lactic acid bacteria in yogurt. 

The purpose of the present study was to investigate the effects of the consumption of an olive fruit polyphenol-enriched yogurt on hematological, physiological and metabolic parameters, blood redox status and body composition in individuals with non-declared pathology, and to study the potential role of olive polyphenols in the microflora of yogurt during fermentation and refrigerated storage (and during the consumption period).

## 2. Experimental Section

### 2.1. Participants

A total of 19 (8 men, 11 women) never-smoking Caucasian adults with non-declared pathology volunteered to participate in this study. All volunteers were informed about the study protocol, filled a medical history questionnaire and signed an informed consent form before participating in the study. Exclusion criteria included a medical history of metabolic or another serious condition that could affect the results of the study, high blood pressure (BP), allergies, lactose intolerance, liver disorders, gastrointestinal disorders (e.g., peptic ulcer), pregnancy, medication use or antioxidant supplementation over the last 6 months. Moreover, volunteers were instructed to avoid lifestyle changes and intense physical activity for at least 2 days before each visit to the laboratory.

### 2.2. Experimental Design

The present study was a randomized, double-blind, placebo-controlled, crossover trial. All participants were randomly assigned to one of two groups, separated by a wash-out period of a week. The experimental condition (EC) group involved daily consumption of yogurt (400 g) enriched with 50 mg of polyphenols for two weeks, whereas the control condition (CC) involved daily consumption of plain yogurt (400 g) not enriched with polyphenols for two weeks. The procedures were in accordance with the 1975 Declaration of Helsinki and approval was received from the University of Thessaly review board (Ethic approval code 1002). This trial was registered at ClinicalTrials.gov [6] as NCT02494739.

The yogurt used in this study was a commercial product of Greek yogurt with 2% fat that was produced from fresh semi-skimmed cow’s milk with the addition of a commercial starter culture of *Lactobacillus bulgaricus* and *Streptococcus thermophiles* (100 g of yogurt contain: 52 kcal; 4.6 g of carbohydrates; 4 g of proteins; 2 g of fat). The yogurts consumed during the EC were supplemented with Medoliva©, a commercial natural product of olive fruit polyphenols encapsulated in maltodextrin, kindly supplied by Polyhealth S.A. Larissa, Greece). The process of production and encapsulation of olive fruit polyphenols has been previously described [5]. This encapsulation process improves olive polyphenol solubility, prevents decolorization of yogurt (which would otherwise occur due to the dark brown color of native olive polyphenol extracts), and facilitates their efficient homogenization into the yogurt matrix and their gradual release into the yogurt (and presumably the gastrointestinal tract). In order to produce the polyphenol-enriched yogurts, 1250 ppm of encapsulated olive fruit polyphenols were added in the milk which was heated to 85 °C for 5 min prior to fermentation and then cooled to 42 °C. After a fermentation period of 8–10 h at 42 ± 1 °C and after the yogurts reached a final pH of 4.3 ± 0.1, the coagulated yogurts were stored at 4 °C for 2 months. 

Participants reported to the laboratory five times. During the first visit, they were informed about the study protocol, filled out a medical history questionnaire and signed an informed consent form. They were also instructed to record their diet and physical activity, in as much detail as possible, for 2 days before their next visit, and follow the same diet and level of physical activity for 2 days before each next visit. Nutrient analysis of the two-day diet records of the participants is presented in Table 1. All physiological measurements were conducted and blood samples were collected in the morning after an overnight fast, before and after the completion of each condition.

### 2.3. Blood Collection and Handling

Blood samples were collected from all participants before and after the completion of each condition. The blood samples (15 mL each) were drawn from a forearm vein, after an overnight fast, and they were then handled as follows:

- *Whole blood*: A portion of blood was collected into EDTA (ethylenediamine tetra acetic acid) tubes for the determination of hematological parameters (complete blood count—CBC) and erythrocyte sedimentation rate (ESR) on the day of blood collection.
- *Plasma*: Another portion of blood was collected into separate tubes containing 20 μL EDTA per 1 mL of blood and centrifuged at 1370× *g* for 10 min at 4 °C. The supernatant (plasma) was transferred into Eppendorf™ tubes (Eppendorf AG, Hamburg, Germany) and they were stored at −80 °C for later determination of total antioxidant capacity (TAC), thiobarbituric acid reactive substances (TBARS) levels and protein carbonyls (PC).
- *Red blood cell lysate*: After plasma collection, a thin grey layer consisting of platelets and white blood cells was also removed in order to obtain the red blood cells. Packed red blood cells were then diluted with distilled water (1:1 *v*/*v*), vortexed vigorously, and centrifuged at 4000× *g* for 15 min at 4 °C. The supernatant was transferred into Eppendorf tubes^®^ and stored at −80 °C for later determination of reduced glutathione (GSH) and catalase activity levels.
- *Serum*: Another portion of blood was collected into separate tubes containing clot activator, left at room temperature for 20 min to clot, and centrifuged at 1370× *g* for 10 min at 4 °C. The supernatant (serum) was transferred into Eppendorf™ tubes and they were stored at −80 °C for later determination of total cholesterol, high density lipoprotein (HDL) cholesterol and triglycerides levels.

Each variable was analyzed in duplicates on the same day. Samples underwent only one freeze–thaw cycle.

### 2.4. Methods

*Assays in whole blood*: CBC was measured with a Mythic 18 (Orphée S.A., Geneva, Switzerland) automated analyzer and ESR was measured by the Wintrobe method.

*Assays in plasma*: In particular, a slightly modified assay of Keles *et al.* [7] was used for thiobarbituric acid-reactive substance (TBARS) determination, as previously described [8]. Calculation of TBARS concentration was based on the molar extinction coefficient of malondialdehyde. The intra-assay percent coefficient of variability for TBARS was 6.5%. The determination of PC was based on the method of Patsoukis *et al.* [9]. Calculation of PC concentration was based on the molar extinction coefficient of dinitrophenylhydrazine. The intra-assay percent coefficient of variability for PC was 4.4% [9]. The determination of TAC was based on the method of Janaszewska and Bartosz [10]. TAC is presented as mmol of DPPH/L plasma reduced to 2,2-diphenyl-1-picrylhydrazine (DPPH:H) by the antioxidants of plasma.

*Assays in red blood cell lysate*: GSH determination was based on the method of Reddy *et al.* [11]. The intra-assay percent coefficient of variability for GSH was 4.2%. For catalase activity determination, the method of Aebi [12] was used. The intra-assay percent coefficient of variability for catalase was 3.8%.

*Assays in serum*: Glucose was measured photometrically in a Clinical Chemistry Analyzer Z 1145 (Zafiropoulos Diagnostica, Athens, Greece) by commercially available kits (Zafiropoulos, Athens, Greece) with the enzymatic colorimetric method GOD/POD/PAP. The intra-assay percent coefficient of variability for glucose was 1.35%. Total cholesterol was also measured photometrically in a Clinical Chemistry Analyzer Z 1145 (Zafiropoulos Diagnostica, Athens, Greece) by commercially available kits (Zafiropoulos, Athens, Greece) with the enzymatic colorimetric method CHOD-PAP. The intra-assay percent coefficient of variability for total cholesterol was 1.5%. High density lipoprotein (HDL) cholesterol was measured photometrically in a Clinical Chemistry Analyzer Z 1145 (Zafiropoulos Diagnostica, Athens, Greece) by commercially available kits (Zafiropoulos, Athens, Greece) with phosphotungstic acid-MgCl_2_ precipitation. The intra-assay percent coefficient of variability for HDL cholesterol was <1%. Triglycerides were measured photometrically in a Clinical Chemistry Analyzer Z 1145 (Zafiropoulos Diagnostica, Athens, Greece) with commercially available kits (Zafiropoulos, Athens, Greece) by the enzymatic chromatometric method GPO-PAP. The intra-assay percent coefficient of variability for triglycerides was 1.56%. Uric acid (UA) was measured photometrically in a Clinical Chemistry Analyzer Z 1145 (Zafiropoulos Diagnostica, Athens, Greece) with commercially available kits (Zafiropoulos, Athens, Greece) by chromatometric assay. The intra-assay percent coefficient of variability for UA was 1.3%.

Low density lipoprotein (LDL) cholesterol was estimated using the Friedewald equation [13].

*Anthropometric measurements*: Standing height was measured to the nearest 0.5 cm (Stadiometer 208; Seca, Birmingham, UK). Body mass (measured to the nearest 0.1 kg) and body fat percentage were measured with a Tanita Body Fat Monitor/Scale TBF-521 (Tanita, Inc., Arlington Heights, IL, USA). During these measurements, participants were lightly dressed and barefoot.

*Physiological measurements*: BP was measured in a sitting position with a manual sphygmomanometer (FC-101 Aneroid Sphygmomanometer; Focal Corporation, Kashiwa, Japan). Mean arterial pressure (MAP) was calculated by the following Equation (1):

MAP = ((2 × diastolic BP) + systolic BP)/3(1)

*Measurement of total titratable acidity of yogurt*: During the fermentation period and the storage period the total acidity, expressed as lactic acid concentration (g/L), was measured after dilution of 10 g yogurt to 30 mL distilled water, and an addition of phenolphthalein and titration with 0.1 N NaOH until a permanent change of color from white to pink. The concentration of lactic acid (g/L) was equal to 0.09 times the volume (mL) of the 0.1 N NaOH consumed during titration with phenolopthalein. 

*Microbiological analysis of yogurt*: The most important indices of yogurt microflora were measured during the fermentation and the storage period (2 months). The population of yeasts and molds was estimated after inoculation of the sample (after appropriate dilutions) to Potato Dextrose agar (NEOGEN) and incubation at 22 °C for 5 days. The total lactic acid bacteria (LAB) were counted in MRS agar (OXOID) after incubation at 30 °C for 5 days, while *Lactobacillus bulgaricus* was inoculated in Lactobacillus Selective agar (NEOGEN) and incubated at 37 °C for 3 days, and *Streptococcus thermophillus* was cultivated in M17 agar (OXOID) and incubated at 37 °C for 3 days. Coliforms were counted in a double layer of Violet Red Bile agar (NEOGEN) after incubation at 37 °C for 24 h, and *Staphylococcus aureus* was measured after spreading the inoculum on Baird Parker agar supplemented with egg yolk tellurite (OXOID) and incubating at 37 °C for 2 days. The microbial populations were analyzed in triplicate and the average values are depicted here.

### 2.5. Statistical Methods

Data are presented as mean ± SEM. Two-way repeated measures analysis of variance (ANOVA) was conducted to analyze the data. If a significant interaction was obtained, pairwise comparisons were performed through simple contrasts and simple main effects analysis using the Bonferroni test method. The level of statistical significance was set at *p* < 0.05. The statistical program used was SPSS version 18.0 (SPSS Inc., Chicago, IL, USA).

## 3. Results

There were three drop-outs; one subject dropped out of the study due to illness (viral gastroenteritis), one subject due to non-compliance (she claimed that two yogurts/day was too much), and another subject because he did not like the flavor of the yogurt. Finally, 16 participants (6 men, 10 women; age: 36.3 ± 4.1 years) completed all two arms of the study protocol. No significant (*p* > 0.05) difference between conditions in baseline values of any parameter tested was observed.

Body weight, body mass index (BMI), hip circumference and systolic BP values decreased significantly (*p* < 0.05) following the two-week consumption of yogurt regardless of condition (Table 2).

No significant (*p* > 0.05) change in CBC parameters was observed following the two-week consumption of Greek yogurt regardless of condition (Table 3).

Regarding the metabolic parameters, a decrease that approached significance (*p* = 0.06) in LDL cholesterol following 2 weeks of polyphenol-containing yogurt consumption was observed (Table 4). No significant differences were observed in glucose, total cholesterol, HDL cholesterol, triglycerides and uric acid.

No change in GSH, catalase activity, PC and TAC levels was observed. A significant (*p* < 0.05) decrease in TBARS levels following 2 weeks of polyphenol-containing yogurt consumption was observed (Table 5).

**Figure 1:**
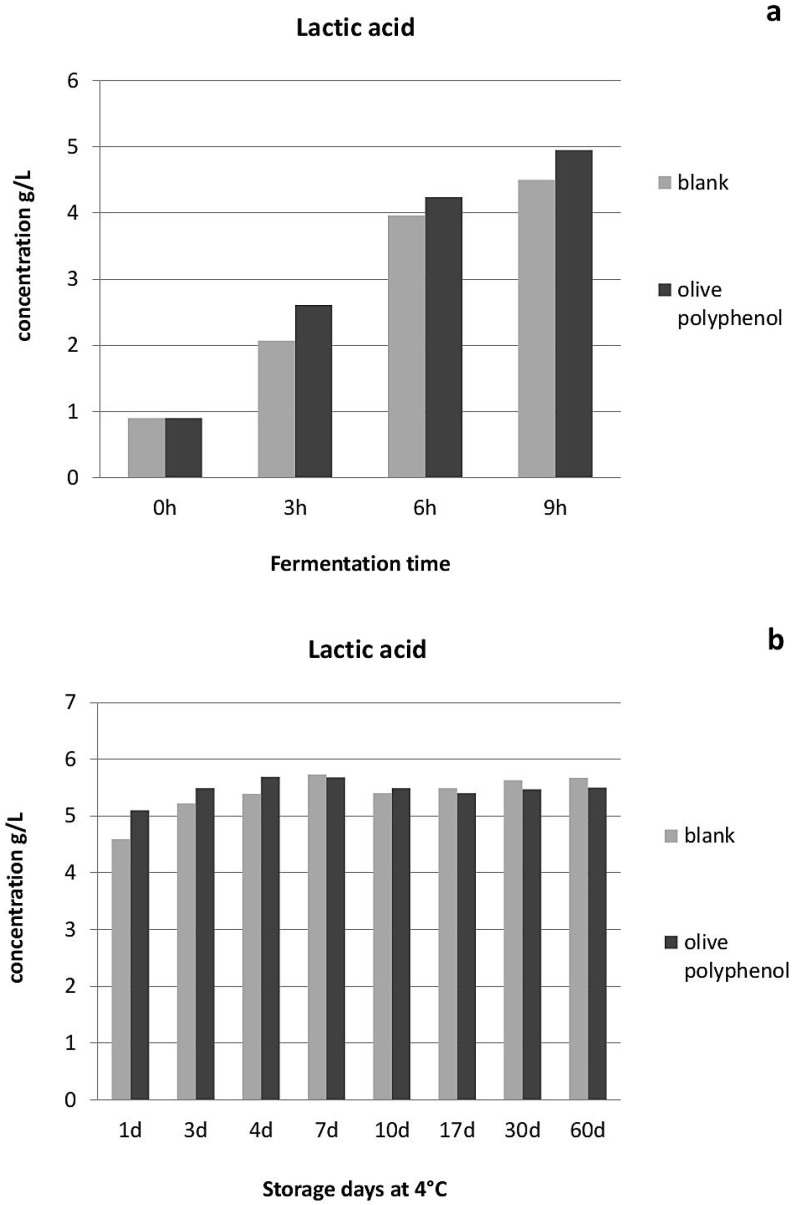
Total acidity (expressed as g/L of lactic acid concentration) during fermentation (**a**) and refrigerated storage (**b**) of yogurts with or without addition of 1250 ppm of olive polyphenols.

**Figure 2:**
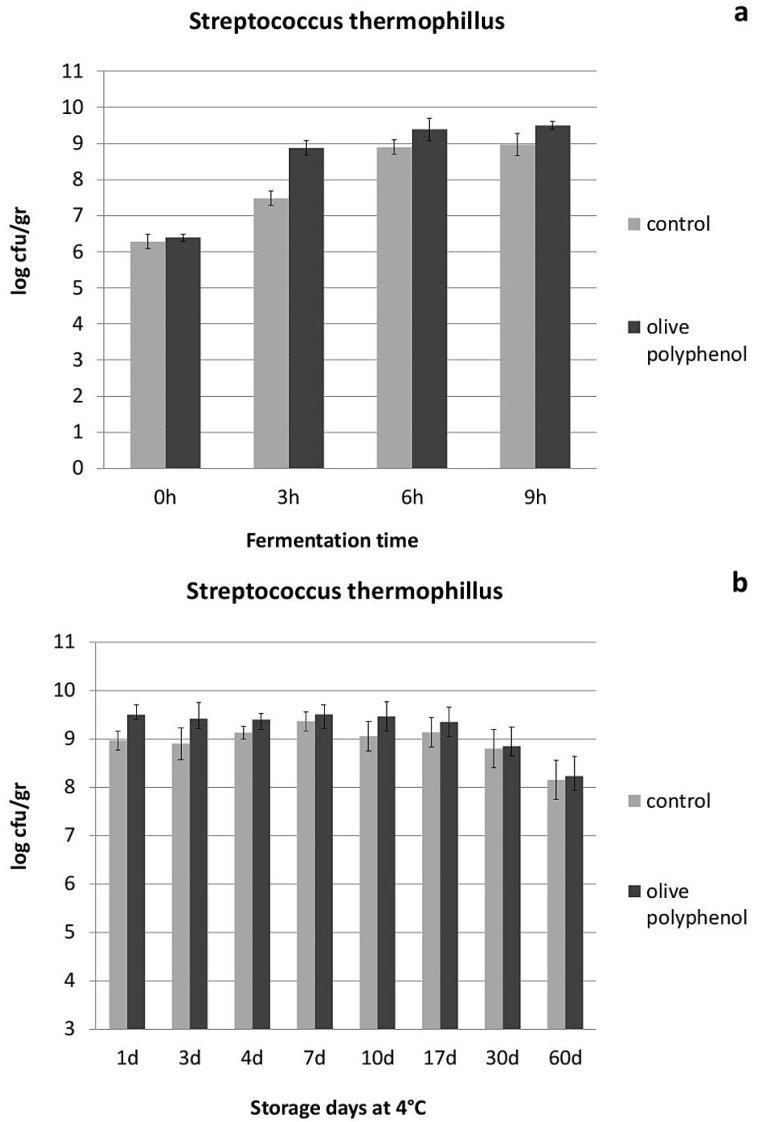
Population of *Streptococcus thermophillus* during fermentation (**a**) and refrigerated storage (**b**) of yogurts with or without addition of 1250 ppm of olive polyphenols.

**Figure 3:**
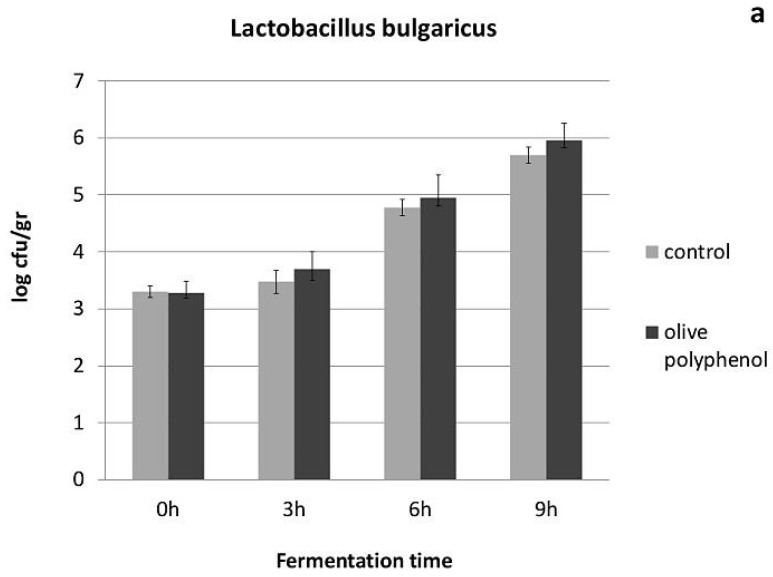

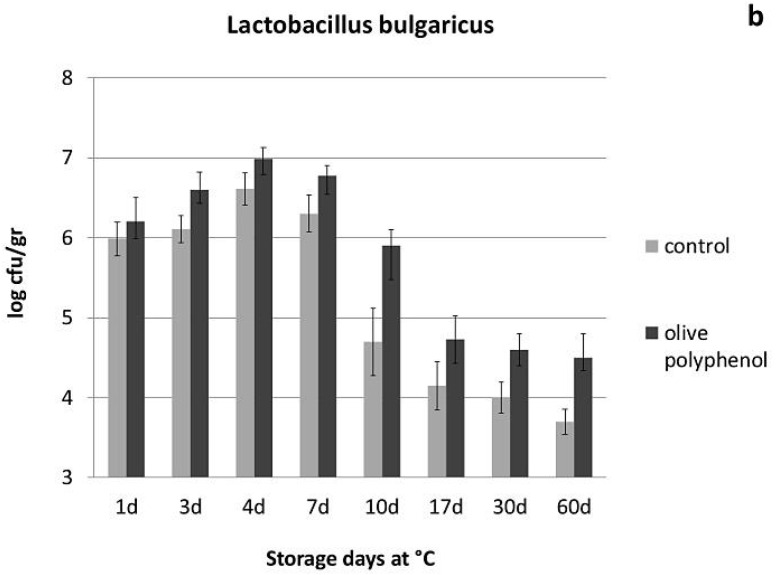
Population of *Lactobacillus bulgaricus* during fermentation (**a**) and refrigerated storage (**b**) of yogurts with or without addition of 1250 ppm of olive polyphenols.

**Figure 4:**
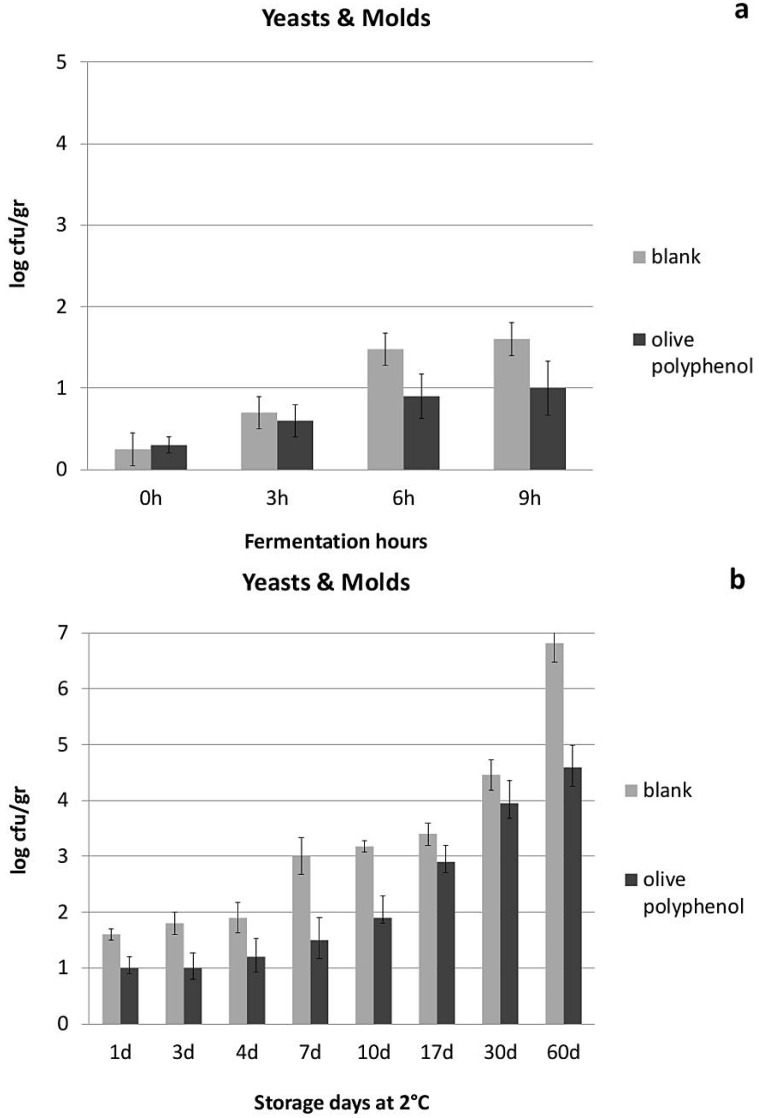
Population of *Lactobacillus bulgaricus* during fermentation (**a**) and refrigerated storage (**b**) of yogurts with or without addition of 1250 ppm of olive polyphenols.

With regard to yogurt physicochemical and microbiological characteristics, yogurt acidity seemed to be influenced by the addition of olive polyphenols, especially during the first hours of fermentation and the first 4 days of storage (Figure 1). This is apparently due to the higher growth of lactic acid bacteria achieved after the addition of olive polyphenols (Figure 2 and Figure 3), especially during the fermentation period. The population of *Streptococcus thermophillus* is higher in the presence of olive polyphenols by 0.4–1.3 log cfu/g in comparison to the control during fermentation, but this difference is less significant between the two types of yogurt after the 3rd day of storage (Figure 2). No growth of *S. thermophillus* took place after the 7th day of storage at 4 °C; however, the population of the *S. thermophillus* remained somewhat higher in the polyphenol-enriched yogurt during the first 17 days of storage, after which the overall population started to decline. However, *Lactobacillus bulgaricus*, which generally attained a lower population during fermentation and storage compared to *S. thermophillus*, achieved a significantly higher growth or survival in the polyphenol-enriched yogurts not only during fermentation, but also throughout the storage period where a 0.2–1.2 log cfu/g difference exists between the control and the polyphenol-enriched yogurts (Figure 3). Conversely, yeast and molds, which are present practically only after the end of the fermentation period and grow exponentially during refrigerated storage, are significantly reduced (by 0.6–2.2 log cfu/g) during storage in the yogurts containing olive polyphenols compared to the control (Figure 4). Microbial safety indicators such as coliforms and *Staphylococcus aureus* were not present in detectable levels in any of the tested samples, either during fermentation or during storage.

## 4. Discussion

Bioactive foods and components have been gaining a lot of attention over the last few years. Observational and experimental studies have extensively investigated the beneficial effects of yogurt consumption on various health parameters in both healthy and unhealthy populations [14,15]. At the same time, there is a large body of evidence suggesting that polyphenols have various beneficial health effects. To the authors’ knowledge, this investigation is the first one examining the possible combined beneficial effects of polyphenols and yogurt in individuals with non-declared pathology.

A significant decrease in systolic BP following two-week consumption of yogurt was observed. Changes in lifestyle, especially diet, can have beneficial effects on risk factors of cardiovascular diseases (CVD), including BP. As part of a healthy diet, consumption of low-fat dairy products can result in reduced BP in both normotensive and hypertensive individuals [16], even when not accompanied by a decrease in body weight [17]. This beneficial effect of low-fat dairy products could be attributed to their high calcium content [18,19] and to other components [20,21]. Moreover, consumption of foods with high polyphenol content has also been shown to cause favorable changes in several risk factors of CVD, including decreased blood BP [22]. However, in the present study, we observed a similar decrease in systolic BP after consumption of both polyphenol-enriched and plain yogurt. Therefore, it could be hypothesized that since added polyphenols did not enhance this effect of yogurt, other components such as calcium could have contributed to decreased systolic BP.

There was a trend for significant (*p* = 0.06) decrease in LDL cholesterol following two-week consumption of polyphenol-enriched yogurt. LDL cholesterol is another risk factor of CVD that observational and experimental studies have shown to be negatively associated with dairy product consumption [14,23]. Moreover, intervention studies have shown that consumption of polyphenol-rich foods can reduce LDL cholesterol levels [24]. The observed result of the present study is more likely attributable to polyphenols added to the yogurt, which were derived from olive fruit. This is in accordance with previous intervention studies that have shown that olive polyphenols may have beneficial effects on LDL cholesterol levels [25]. Therefore, we suggest that 2 weeks of yogurt consumption can result in reductions in LDL cholesterol that can be attributed to the olive fruit polyphenol found in the yogurt.

Regarding indices of blood redox status, no significant (*p* > 0.05) change in GSH, catalase activity and PC levels was observed, whereas TBARS levels were significantly (*p* < 0.05) decreased after a two-week consumption of polyphenol-containing yogurt. Malondialdehyde (MDA), as measured by TBARS assay, is an indicator of lipid peroxidation, a process that results in the destruction of membrane lipids and, subsequently, cell death. Some previous studies have shown that olive polyphenols may have antioxidant effects that protect against lipid perodixation [25], and some others have indicated that MDA levels are increased in hyperlipidemic individuals [26,27]. In addition, rich in polyphenols pomegranate juice consumption for 15 days, reduces lipid peroxidation in human blood [28]. Another study has shown that a special carbohydrate-protein bar and a tomato juice supplementation in ultra-marathon runners for a two-month period, significantly improved the oxidative status of the subjects [29]. Therefore, decrease in TBARS levels may be associated with decreased LDL cholesterol levels observed after the 2-week consumption of polyphenol-enriched yogurt. Although polyphenols are thought to have strong antioxidant properties, no change in other indices of blood redox status after yogurt consumption was observed. The present results indicate that two-week consumption of polyphenol-containing yogurt may result in reduced lipid peroxidation and, therefore, may exert cardiovascular protection due to its high olive fruit polyphenol content.

Body weight, BMI and hip circumference decreased significantly following two-week consumption of yogurt with or without polyphenols. There are some data indicating that frequent yogurt consumption may influence energy intake regulation due to its high protein, low fat, low sugar and high calcium content [30]. In the present study, it was observed that 2 weeks of yogurt consumption was sufficient to promote body weight loss in both men and women; however, no change in body composition was evident. Meta-analyses have shown that consumption of yogurt is associated with favorable changes in body composition only when accompanied by energy intake restriction [31,32]. The present intervention did not involve energy intake restriction. Factors such as reduced hunger, increased fullness and healthier food choices due to yogurt consumption during intervention could potentially explain the significant body weight loss. Therefore, more research on the effect of yogurt consumption in such factors may be more enlightening. Moreover, it is thought that hip circumference has an inverse association with dyslipidemia [33], type 2 diabetes [34] and cardiovascular disease [35] when waist circumference is taken into account.

As concerns the physicochemical and microbiological characteristics of yogurt after the addition of olive polyphenols, it appears that at 1250 ppm these encapsulated polyphenols can increase the concentration and the rate of accumulation of lactic acid, which is a desirable quality trait in yogurt, since it helps prevent potential spoilage during fermentation, and also indicates a higher rate of metabolism in LAB. This observation is in agreement with the results of Petrotos *et al.* [5] showing that the addition of 500 ppm of free or starch-encapsulated olive polyphenols significantly reduced the pH and thus increased the acidification rate in yogurt during the first 24 h (fermentation period), and the study of Giavasis *et al.* [4], reporting an increased accumulation of lactic acid by different lactobacilli (including *L. bulgaricus*) in a synthetic medium containing olive polyphenols. This increase in total yogurt acidity is evidently a result of the higher growth of lactic acid bacteria of the starter culture in the polyphenol-enriched yogurts. 

Interestingly, both *S. thermophillus* and *L. bulgaricus* seem to be stimulated by the presence of olive polyphenols in yogurt and, not only a higher growth rate during fermentation, but also a higher survival rate during storage is observed, especially for *L. bulgaricus* in the polyphenol-enriched yogurts. On the contrary, yeast and molds which are the main spoilage microorganisms in yogurt seem to be partly inhibited after the addition of olive polyphenols (probably via the induced growth of LAB). Apart from the significant technological implications of these observations (*i.e.*, faster acidification and coagulation rate, shorter production time, increased storability), the presence and the population of these beneficial lactic acid bacteria may play an important role in both digestion as well as lipid metabolism, obesity and atherosclerosis [36]. *Lactobacillus* species are known for being able (along with Bifidobacteria) to modify gut microflora, and have been shown to influence the levels of key enzymes of metabolism, such as bile salt hydrolase, azoreductase, β-glucuronidase and the proportions of unconjugated/conjugated bile acids [37]. Jin *et al.* [38] observed an increased amylase and decreased *β*-glucuronidase and *β*-glucosidase activity in the intestines of chicken fed with a mixture of lactobacilli. Also, the potential cholesterol-lowering effect of different *Lactobacillus* species has been described by different studies [39,40]. Chiu *et al.* [41] showed that the administration of fermented milk containing different species of lactobacilli could reduce cholesterol levels in serum and liver of rats by 13% to 30% depending on the species used. Therefore, it is possible that some of the biochemical/physiological functions of the polyphenol-enriched yogurts found in this study, such as the reduced LDL cholesterol levels (or the improved body weight loss), may be at least partly linked to the higher populations and the enhanced metabolism of lactic acid bacteria in yogurt, as a result of the addition of olive polyphenols.

## 5. Conclusions

The results of the present study indicate that the polyphenol-enriched yogurt consumption may help individuals with non-declared pathology reduce body weight and systolic BP, while the added olive fruit polyphenols (50 mg per day) may decrease LDL cholesterol and lipid peroxidation levels in individuals with non-declared pathology. They also enhance the growth of lactic acid bacteria in yogurt, which may have several beneficial consequences in lipid metabolism. These findings are important as they suggest that the addition of olive polyphenols may reverse potentially undesirable effects of conventional dairy product consumption (such as the increase of LDL cholesterol, due to their content of saturated fats). Consumption of yogurt containing 50 mg of olive fruit polyphenols over a longer period of time may induce greater favorable changes in metabolic parameters and indices of redox status in both healthy and unhealthy populations. Even though the present study provides some promising results with regard to cardiovascular protection following polyphenol consumption in yogurt, more research on clinical populations is needed in this area.

## Figures and Tables

**Table 1:** Nutrient analysis of the two-day diet records of the participants (Mean ± SEM).

	Group A	Group B
Energy (kcal)	1965.5 ± 120.3	1899.2 ± 196.3
Carbohydrate (g)	231.9 ± 12.3	225.3 ± 42.4
Protein (g)	91.5 ± 10.3	69.2 ± 7.5
Fat (g)	87.7 ± 6.0	77.0 ± 7.0
Fiber (g)	14.3 ± 1.2	18.4 ± 6.2

Group A is for participants that were first assigned to the experimental condition, whereas group B is for participants that were first assigned to the control condition.

**Table 2:** Anthropometric and physiological parameters following plain (CC) and polyphenol-enriched (EC) yogurt consumption (Mean ± SEM).

Parameter	EC		CC	
	Pre	Post	Pre	Post
Body weight (kg)	70.0 ± 3.3	69.7 ± 3.4 *	70.4 ± 3.4	69.5 ± 3.3 *
BMI (kg/m^2^)	24.5 ± 0.6	24.3 ± 0.7 *	24.6 ± 0.7	24.3 ± 0.7 *
Body Fat (%)	26.9 ± 1.6	26.9 ± 1.6	27.5 ± 1.6	27.1 ± 1.7
Waist (cm)	80.4 ± 2.2	80.2 ± 2.3	80.3 ± 2.2	79.8 ± 2.1
Hip (cm)	101.5 ± 1.3	101.0 ± 1.4 *	101.9 ± 1.4	101.4 ± 1.5 *
WHR	0.793 ± 0.02	0.792 ± 0.02	0.779 ± 0.02	0.779 ± 0.01
SBP (mm Hg)	108.2 ± 2.1	106.7 ± 2.3 *	110.3 ± 2.8	105.8 ± 1.7 *
DBP (mm Hg)	70.4 ± 1.8	70.6 ± 1.9	70.4 ± 2.2	69.4 ± 1.3
MAP (mm Hg)	83.0 ± 1.8	82.7 ± 2.0	83.7 ± 2.3	81.5 ± 1.4

* Significant (*p* < 0.05) difference from pre-value at the same condition. ΒΜΙ: Body mass index, WHR: Waist to hip ratio, SBP: Systolic blood pressure, DBP: Diastolic blood pressure, MAP: Mean arterial pressure.

**Table 3:** Complete blood count parameters following polyphenol-enriched (EC) and plain (CC) yogurt consumption (Mean ± SEM).

Index	EC		CC		
	Pre	Post	Pre	Post	Normal Rage
WBC (10^3^/μL)	5.98 ± 0.3	5.93 ± 0.3	5.83 ± 0.3	5.91 ± 0.3	4.0–12.0
RBC (10^6^/μL)	4.59 ± 0.1	4.58 ± 0.1	4.52 ± 0.1	4.58 ± 0.1	4.00–6.20
HGB (g/dL)	12.86 ± 0.4	12.49 ± 0.4	12.63 ± 0.4	12.61 ± 0.4	11.00–18.00
Hct (%)	42.45 ± 1.1	41.73 ± 1.3	41.53 ± 1.2	41.52 ± 1.1	35.00–55.00
Platelets (10^3^/L)	268 ± 17	271 ± 14.8	249 ± 14.8	271 ± 17.5	150–400

WBC: White blood cells; RBC: Red blood cells; HGB: Hemoglobin; Hct: Hematocrit.

**Table 4:** Metabolic parameters following polyphenol-enriched (EC) and plain (CC) yogurt consumption (Mean ± SEM).

Index	EC		CC		
	Pre	Post	Pre	Post	Normal Rage
Glucose (mM)	4.92 ± 0.14	5.04 ± 0.12	5.10 ± 0.09	5.16 ± 0.08	4.2–6.4
T-Chol (mM)	4.50 ± 0.20	4.39 ± 0.17	4.44 ± 0.14	4.54 ± 0.18	<5.7
LDL-Chol (mM)	2.48 ± 0.16	2.32 ± 0.14 *	2.39 ± 0.10	2.48 ± 0.15	<4.1
HDL-Chol (mM)	1.55 ± 0.08	1.54 ± 0.10	1.52 ± 0.10	1.56 ± 0.10	1.0–3.9
TG (mM)	1.02 ± 0.12	1.16 ± 0.10	1.16 ± 0.14	1.10 ± 0.10	<1.7
UA (μmol/L)	321 ± 19	305 ± 21	301 ± 19	305 ± 17	200–420 (men)
					140–340 (women)

* Significant (*p* = 0.06) difference from pre-value at the same condition. T-Chol: Total cholesterol; LDL-Chol: Low density lipoprotein; HDL-Chol: High density lipoprotein; TG: Triglycerides; UA: Uric acid.

**Table 5:** Indices of redox status following polyphenol-enriched (EC) and plain (CC) yogurt consumption (Mean ± SEM).

Index	EC		CC	
	Pre	Post	Pre	Post
GSH (μmol/g Hb)	2.75 ± 0.20	3.63 ± 0.36	2.90 ± 0.19	3.63 ± 0.4
Catalase (U/g Hb)	218 ± 5.5	222 ± 6.3	218 ± 5.5	233 ± 7.0
TAC (mmol DPPH/L)	0.86 ± 0.02	0.89 ± 0.02	0.91 ± 0.02	0.88 ± 0.02
PC (nmol/mg protein)	0.50 ± 0.02	0.49 ± 0.01	0.49 ± 0.02	0.48 ± 0.02
TBARS (μmol/L)	5.0 ± 0.02	4.1 ± 0.02 *	4.7 ± 0.01	4.4 ± 0.02

* A significant (*p* < 0.05) difference after 2 weeks of polyphenol-containing yogurt consumption compared to baseline. GSH: Reduced glutathione; TAC: Total antioxidant capacity; PC: Protein carbonyls; TBARS: Thiobarbituric acid reactive substances.
